# Understanding Sustainability in Operating Theaters: An Ethnographic Study to Determine Drivers of Unsustainable Behaviors

**DOI:** 10.1097/AS9.0000000000000635

**Published:** 2026-01-05

**Authors:** Aws Almukhtar, Carys Batcup, Sadhana Jagannath, Daniel R. Leff, Talya Porat, Gaby Judah, Pelin Demirel

**Affiliations:** From the * Department of General Surgery, Imperial College Healthcare NHS Trust, London, United Kingdom; † Department of Surgery and Cancer, Imperial College London, London, United Kingdom; ‡ Dyson School of Design Engineering, Imperial College London, London, United Kingdom; § Faculty of Social and Behavioural Sciences, University of Amsterdam, Amsterdam, the Netherlands; ‖ The Breast Unit, Imperial College Healthcare NHS Trust, Charing Cross Hospital, London, United Kingdom.

**Keywords:** sustainability, surgery, determinants of behaviors

## Abstract

**Background:**

Climate change is the biggest threat to human health. Paradoxically, the healthcare sector is a major contributor to climate change, and operating theaters are among the highest sources of emissions. Unsustainable practices are actions that compromise environmental, social, and financial sustainability, leading to unnecessary resource use, avoidable harm to the wider population, and reduced ability to provide effective healthcare in the future. Drivers of unsustainable practices and barriers to sustainability in practice (a top priority identified by the James Lind Alliance Priority Setting Partnership) are unexplored, hindering interventions that can help meet net-zero targets within healthcare. We conducted the first known ethnographic study to investigate behaviors related to sustainability in operating theaters, and their influences on those behaviors to inform the design of effective behavior change interventions.

**Methods:**

Nonparticipant ethnographic observations with opportunistic discussions in elective general surgical operating theaters were conducted between June and December 2023 at 2 university hospitals in Central London. Data were collected until saturation using a template developed during the initial observations. Inductive thematic analysis was conducted, with subthemes (influences) deductively mapped to the Theoretical Domains Framework.

**Results:**

Twenty-six procedures were observed (42 hours). Unsustainable behaviors included: (1) unnecessary and inappropriate glove use, potentially compromising safety (average 8–10 pairs per operation), (2) incorrect waste disposal, (3) unnecessary package opening, and (4) energy waste. Thematic analysis generated 6 themes and 16 influences (mapped to 9 Theoretical Domains Framework domains). Key themes were that sustainable practices are “infrequent and inconsistent” due to limited awareness (Knowledge) and low environmental concerns (Memory, Attention, and Decision Processes). Unsustainable behaviors were “habitual” and performed automatically (Lack of Attention). Drivers of unsustainable practices were: “Precaution” (Emotions, Beliefs About Consequences); “Efficiency” (Goals); “Past experiences” (Emotions and Social influences); and the “Physical environment” (Environmental Context and Resources). “Leadership” (Social Influences) was a driver of sustainable practices.

**Conclusions:**

This study identified widespread unsustainable culture and practices in operating theaters that compromise patient safety, and financial and environmental sustainability. It provides a nuanced understanding of contextual factors and their drivers, such as the strong impact of habit, knowledge, and the striving for efficiency, highlighting the need for both bottom-up engagement and top-down prioritization. The study provides a foundation for designing targeted interventions that integrate education, leadership engagement, and environmental restructuring to embed sustainability into routine surgical practice while ensuring patient safety and operational efficiency.

## INTRODUCTION

Climate change is arguably the “most significant threat” to human health in the 21st Century.^1^ Paradoxically, the healthcare sector is a major contributor to greenhouse gas emissions (GHGe) and the UK National Health Service (NHS) is responsible for approximately 4.0–5.9% of national GHGe, and is a large contributor of waste.^2^ In healthcare, surgery is consistently ranked among the top 3 sources of GHGe, with operating theaters (OTs) being significant contributors owing to high energy demands and waste generation.^2–5^ It is estimated that a single operation generates up to 814 kg of carbon dioxide equivalent, comparable to driving 2273 miles in an average petrol car.^4^

Several studies have outlined interventions to reduce the environmental impact of OTs by changing the practices of operating staff.^6–12^ Additionally, the Royal Colleges’ Intercollegiate “Green Theater Checklist” outlines sustainable practices that would reduce the environmental impact of surgery.^13^ However, the designing of behavioral interventions and the adoption of sustainable practices remain challenging due to the inadequate understanding of the determinants of sustainability practices in OTs.^14^ Although certain studies have highlighted important determinants of sustainability in OTs, such as knowledge and infrastructure,^14^ there are currently no observational studies examining these determinants in practice. Consequently, the nuanced drivers of unsustainable behaviors such as contextual factors, unconscious practices, and social dynamics, are presently unexplored.^15^ Our recent systematic review highlighted the need for nuanced qualitative insights into the influences and contextual factors that underpin sustainability practices in OTs.^14^ This was also identified as the third-highest ranked research priority in the James Lind Alliance Priority Setting Partnership for sustainable perioperative practices.^16^

This study represents the first known ethnographic study of sustainable behaviors in OTs. Sustainability in OTs is a complex and multifaceted issue shaped by individual behaviors, organizational structures, and wider systemic policies. To address this complexity, we employed an ethnographic approach that situates behaviors within their real-world context, incorporates reflexivity, and engages both clinical and nonclinical researchers. Ethnography is a qualitative and immersive research methodology in which researchers use observations, interviews, and field notes to study and identify determinants of behavior, including contextual factors, perspectives of various professional groups, decision-making processes, and the interplay between groups as they work as a team, providing a deep nuanced understanding of the relevant behaviors in the natural setting.^17–20^ Ethnography can also highlight more subconscious or habitual behaviors of which individuals may not be aware, and as such could be missed by interview and survey studies.^14^ In this study, sustainable behaviors refer to actions performed by individuals that lessen the environmental impact of surgery while maintaining patient safety and clinical efficacy, whereas unsustainable behaviors contribute to environmental harm without clinical justification.^12^

This study applies the Theoretical Domains Framework (TDF), a widely recognized and employed framework in behavioral science, to understand the determinants of behavior.^21,22^ The TDF was developed through a collaboration between behavioral scientists and implementation researchers, who identified relevant theories and organized their constructs into domains, with the goal of providing a comprehensive, theory-based approach to identifying the determinants of behaviors.^21^ The TDF may facilitate a comprehensive examination of the key influences on sustainability behaviors and can be combined with other behavior change tools (eg, the Behavior Change Technique Taxonomy^23^ and the Theory and Techniques Tool^24^) to design interventions that are more likely to change behavior. This study aimed to investigate the occurrence and understand the determinants of (un)sustainable behaviors in OTs.

## METHODS

### Qualitative Approach and Researcher Characteristics

Rooted in a critical-realist epistemological framework,^25^ this study employed a nonparticipatory ethnographic research design with opportunistic discussions. The researchers’ experiences and backgrounds allowed them to be both insiders and outsiders in OTs. A.A. is a surgical registrar and an academic researcher who has conducted research on surgical sustainability and worked in OTs. This allowed him to capitalize on the benefits of being an outsider (part-time clinician who is not based at the observation sites), questioning taken-for-granted assumptions, and an insider in the natural setting who understands the processes and is able to unravel the nuances of behaviors.^26^ D.R.L. is a consultant academic surgeon with extensive clinical and nonclinical research experience. S.J., C.B., P.D., T.P., and G.J. are experienced nonclinical researchers. Their relative unfamiliarity with OTs allowed them to act as outsiders and mitigate any presuppositions.

### Context

The research was conducted at a major London NHS Trust (publicly funded, semi-autonomous healthcare organization within the NHS), comprising 3 university hospitals. Observations were made in 4 specialties (general surgery, breast surgery, colorectal surgery, and procedures for chronic pain) to enhance generalizability by providing insights across diverse surgical contexts with generally similar practices and infection prevention protocols. Elective operations were chosen to focus on routine surgical settings and eliminate specific circumstances (eg, where extreme urgency is paramount).^27,28^ Detailed characteristics of the specialities are outlined in Supplemental Table 1, https://links.lww.com/AOSO/A559.

### Sampling Strategy

Purposive sampling was employed in the selection of specialties (as described above) and hospitals (which were chosen given the primary ethnographer’s established familiarity and rapport from his clinical role). This enabled access to various OTs and facilitated an unobtrusive presence, allowing the ethnographer to blend in with the OT staff. Drawing upon the principle of informational power^29^ (taking into account the aim of the study, specificity of the research question, applied theory, and analysis strategy), 25–30 hours of observation were estimated to be sufficient. Sampling was stopped when saturation was reached (when no new themes or meanings were identified).^30^ Data saturation was reached when practices were being repeated, and when no new insights from observed behaviors could be obtained, indicating that sufficient information had been gathered to replicate the study.^31^

### Ethical Approval

This study is part of a larger programme of work aiming to coproduce behavioral interventions to empower environmentally sustainable behaviors in the United Kingdom (IRAS project ID: 322218). Ethical clearance for observations was obtained from the research and ethics committee of the respective hospitals, ensuring compliance with the established ethical standards (RN: 857).

### Consent

Written consent was obtained from the respective OT coordinators, while verbal consent was obtained from the participants at the beginning of observations, and from individuals participating in communication interactions. Verbal consent from the patients was obtained where appropriate; however, since no patient-related data were collected or observed, written consent was not deemed necessary.

### Procedure

Fieldwork was completed between June and December 2023. Observations encompassed all stages of the surgical process, except induction of anesthesia due to logistics and ethical concerns (anesthesia rooms are separate from OTs and hospital policy advises minimizing staff presence at the induction stage of anesthesia to reduce patient anxiety).

The study started with an unstructured investigative approach (grand tour observations^32^), whereby the main ethnographer (A.A.) and the senior authors each observed 1 operating list (a session) (hereafter, “list”). The purpose of grand tour observations is to gain a broad understanding of the research setting, and to identify relevant actors and processes without imposing preconceived ideas.^33,34^ These observations were also used to inform the development (through an iterative process) of a data collection sheet, which was tailored to investigate the drivers of sustainability behaviors in OTs, reflecting the natural flow of activities. Additionally, broad subjects for questioning were discussed and agreed among team members in relation to participants’ perceptions and understanding of sustainability during this phase. Further unstructured questions were employed based on what was observed in those specific contexts.

The uncoded data collection sheet format consisted of (1) a structured section to collect information about the surgical staff and their role, the environment in the theater complex, the sustainability behaviors and their contexts in various perioperative stages, and (2) a larger free-text section. This format enabled qualitative and quantitative assessment of the use of both pre-identified consumables [eg, surgical hats and nonsterile gloves (NSGs)] and non-pre-identified (eg, swabs and overall counts), which were noted in the free-text section. This approach supported systematic capturing of sustainability behaviors and their contextual factors, while allowing flexibility to document further observations (Supplemental Table 1, https://links.lww.com/AOSO/A559).

In common with many institutions around the world, there is no formal policy on double-gloving and the routine use of eye-protection, and they are not standardized practices in our institution.^35^ As such, these practices were examined separately, and were not categorized as universally sustainable or unsustainable (Supplemental Table 3, https://links.lww.com/AOSO/A559).^36–38^ Instead, instances where the staff opted to double-glove or use eye-protection were examined as contrasting practices between individuals in the same procedure, and between the same individuals in different procedures, and were discussed with the participants to identify their behavioral drivers in the specific context, and then within the research team. This approach allowed us to interpret behaviors within their clinical and institutional contexts and to identify the drivers behind them. For these practices, we examined low-risk procedures [procedures which are not: orthopedic or dental surgeries involving sharp structures; emergency and trauma operations; procedures performed at night; procedures where the primary surgeon was a registrar (trainee); or when the preoperative screening identified patients with blood-born infections or carriers of a resistant organism (see Supplemental Table 3, https://links.lww.com/AOSO/A559)].

Instances of equipment and consumables that were unnecessarily used (eg,NSGs) or unused (eg, swabs and sutures) were noted as part of the qualitative field notes. These were recorded as overall counts in comparable operations to illustrate patterns of waste generation, rather than to provide a quantified assessment of environmental impact. This approach aligns with the ethnographic focus on understanding behaviors and contextual influences rather than producing statistical comparisons.

### Analysis

The data collection sheet (which was developed in the early stages of the study) contained organizational categories based on the research question, and served as a tool to ensure the data collection was robust and contextualized.^39^ The analysis was conducted using NVivo software (QSR International, Melbourne, Australia). The data collected from the free-text observations were inductively analyzed utilizing interpretative and descriptive coding. A.A. and C.B. double-coded 50% of the data. These codes were grouped into themes and subthemes to contextualize and describe the influences.^40^ The influences were then deductively mapped (coded) onto the TDF to ensure rich analysis of the data and trustworthy understanding of the determinants. Fereday and Muir-Cochrane^41^ demonstrate the greater rigor that can be achieved in thematic analysis when a combination of inductive and deductive thematic analysis, with the aim of harnessing the advantages of each, was employed. As such, this method has been widely adopted in healthcare behavioral research.

The data and analysis were discussed fortnightly with the research team to ensure agreement of codes amongst the research team. The work has been reported in line with the COnsolidated criteria for REporting Qualitative Research criteria (Supplemental Table 4, https://links.lww.com/AOSO/A559).^42^

## RESULTS

Data collection was concluded when saturation was reached, after 42 hours. Twenty-six elective surgical procedures were observed. Details of specialities and staff observed are outlined in Supplemental Table 2, https://links.lww.com/AOSO/A559. Supplemental Figure 1 https://links.lww.com/AOSO/A559 illustrates and describes the OT complex. The pertinent unsustainable practices were: (1) unnecessary glove use (average 8–10 pairs per operation), (2) incorrect waste segregation and disposal, (3) unnecessary opening of packages, and (4) energy waste.

### Themes and Influences

**FIGURE 1.**
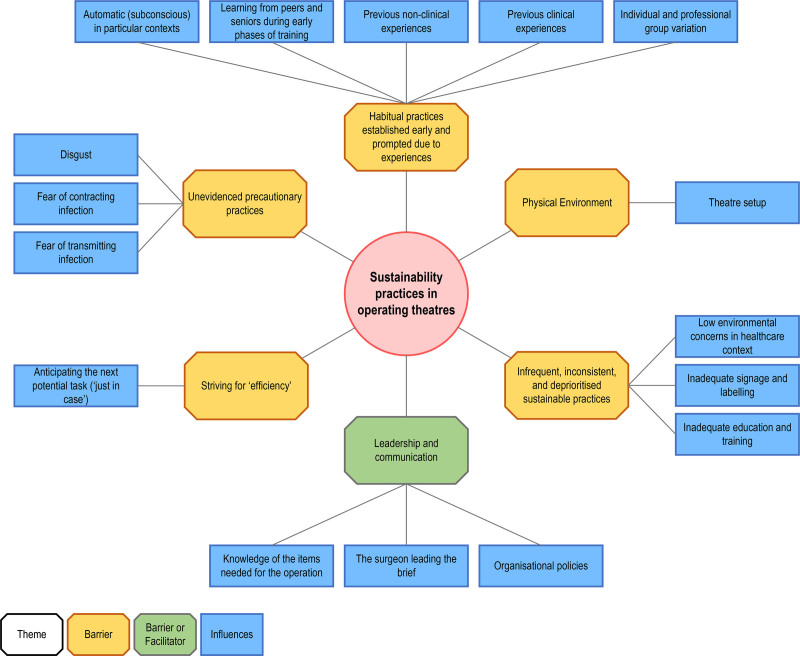
Thematic map of the themes and influences on (un)sustainable practices in OTs. Six themes encompassing 16 influences were identified. Themes comprised of barriers are demonstrated in yellow, while themes containing barriers and facilitators are illustrated in green.

Six themes and 16 influences were identified. The influences were mapped to 9 of the 14 TDF domains (Fig. 1 and Supplemental Table 5, https://links.lww.com/AOSO/A559).

#### Theme 1: Sustainable Practices are Infrequent, Inconsistent, and Deprioritised

**FIGURE 2.**
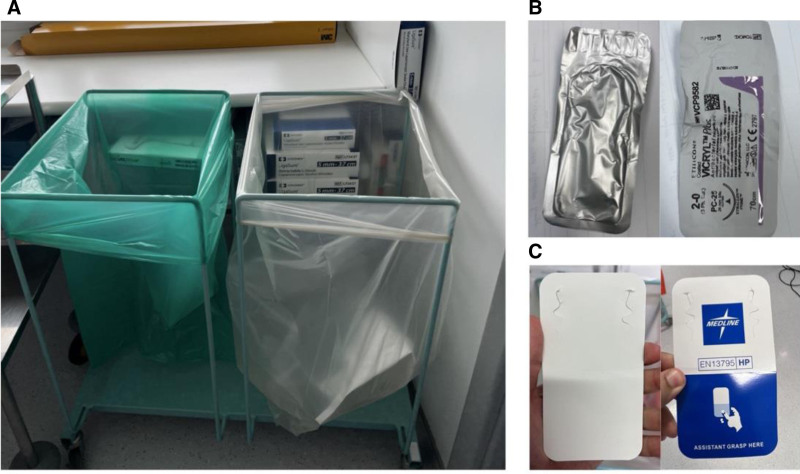
Photographic images demonstrating inadequate labeling and signage. Bins, packages, and paper were inadequately labeled to guide theater staff to the correct recycling and disposal practices. A, Two different bins (Green for paper and transparent for dry mixed recycling) without proper signage. B and C, Different items with unknown recyclability due to inadequate labeling.

The observed sustainability practices were waste segregation and recycling. These practices were inconsistent, and often not performed correctly. Lack of adequate education and training (Knowledge) was an influence; staff felt that they did not receive adequate training and education on waste segregation. While online training was made available, it was not specific to OTs and was not comprehensive or mandatory. Staff reported conflicting sources of information on how to dispose of different items in OTs (eg, the “blue wrap,” used extensively in OTs after sterilization). This was compounded by inadequate signage and labeling of items and packaging (Environmental context and resources). Bins were not labeled, and packages did not have adequate segregation and recycling instructions (Fig. 2). Finally, environmental concerns were not considered as important in the healthcare setting as they would be in day-to-day life (Memory, Attention, and Decision Processes). A Higher Speciality Registrar (SpR) double-gloved for a low-risk operation, when the primary surgeon did not (unsustainable practice; refer to Supplemental Table 3, https://links.lww.com/AOSO/A559). They explained that despite being environmentally conscious and making efforts to be sustainable outside work, they did not feel they should do the same at work, that safety should be the main concern, and that sustainable practices compromised patient care [Observation 1; Surgeon 2 (O1; S2)]. A consultant surgeon explained that the context of work, they “don’t care about the environment” and that surgeons who make conscious efforts to be environmentally sustainable “don’t do their jobs well” (O4; S1).

#### Theme 2: Habitual Practices Established Early and Prompted Due to Experiences

**FIGURE 3.**
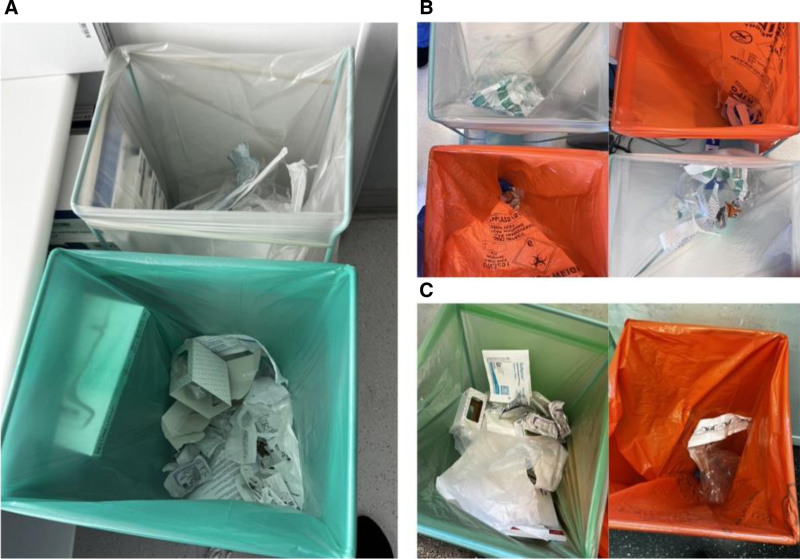
Photographic images demonstrating different items discarded randomly and incorrectly. These were discarded by staff without active thought. For context: paper should be discarded in green bins and film in the white bin as per recycling policy. The orange bin should be reserved for infectious contaminated clinical waste.

Unsustainable practices appear to occur without conscious thought, intention, or awareness. Practices like donning and discarding NSGs and double-gloving seem to be performed habitually, and without active thought. For example, 3 nurses donned NSGs to move equipment and discarded them in the infectious bin whilst conversing amongst themselves (O1; N2; N4; N7). In another instance, 2 nurses donned gloves to use a computer whilst carrying out informal conversations. Neither of these tasks requires gloves. A surgical SpR who double-gloved for all their operations (which were low-risk) explained, “I always double glove; I like to do things the same way every time” (O2; S3). Figure 3 demonstrates examples of instances when OT staff discarded items without paying attention.

These habits were learned during early stages of clinical training from peers and seniors and were prompted and reinforced by lived and heard experiences (Social influences). A nurse and an anesthesia consultant discarding packages explained that they learned waste segregation from watching their peers—“all I know is what others told me.” A nurse who double-gloved unnecessarily for a low-risk procedure explained how their previous experience in orthopedic surgery (high-risk speciality) had shaped and standardized their practices—“I was an orthopedic nurse and in orthopedic, we always wear double gloves as the risk of contamination is high.” (O2; N4). Moreover, past experiences, both clinical and nonclinical, triggered emotional responses reinforcing unsustainable practices (Emotions) and prompted the staff to adopt more risk-averse behaviors, such as unnecessary gloving. A nurse explained that they are fearful and conscious of germs because they previously experienced illness from eating at the university canteen as a student. Consequently, they adopted routine NSG donning for all tasks in theaters for “protection”. They further explained that they “don’t feel comfortable” without NSGs (O6; N1).

Variations between professional groups and individuals of the same group were also observed. Some nurses unnecessarily donned NSGs to prep the OT while others did not. Furthermore, the behavior of other OT staff, or knowledge of sustainable practices, did not influence habitual unsustainable practices, demonstrating the strength of habits as a driver of unsustainable practices. An SpR surgeon double-gloved to assist in an elective laparoscopic cholecystectomy (low-risk), when the consultant surgeon opted to don single-gloves (O5; S1:S2) (see Supplemental Table 5 for more examples https://links.lww.com/AOSO/A559). Nurses were more risk-averse and performed more unsustainable practices in comparison to other clinicians (Social/Professional Role and Identity).

#### Theme 3: Unevidenced Precautionary Practices

Safety and precaution played an influential role in driving staff behaviors in OTs, and unsustainable practices occurred in the absence of evidence or clear institutional guidance. A trainee-surgeon consistently wore surgical masks with eye-protection to assist in procedures where the operating surgeon and other assistants used simple surgical masks explained that they always wear it “just in case there is a blood splatter” (O2; S3). Staff were afraid of contracting infection from contact with patients, and maintained a physical barrier between themselves and patients, even when the risk was equivalent to the risk of contact with the general population (Emotions). For instance, OT staff donned NSGs to push clean patients’ wheelchairs or hold their hands to help them off the bed. An anesthesia nurse said, “You never know if the patient has HIV, you need to always be protected”. When the anesthesia consultant explained that HIV is a blood-borne virus and cannot be transmitted through skin-to-skin contact, the nurse said, “What if I have micro-cuts on my skin?” (O2; AN1; A1).

These practices demonstrated that heightened perception of risk was the default. Subsequent observations that involved patients with known HIV cases were noted to have no apparent difference in behaviors of NSG use, confirming the persistently high-risk perceptions in OTs (O7 and O8). OT staff avoided direct contact with patients and treated NSGs as physical barriers to perform activities that they otherwise may be “disgusted” to perform (Emotions). This included donning NSGs to shave the clean abdomen of an elective patient who underwent screening and decontamination, or wearing NSGs to hold clean long forceps and using them to dispose of soiled swabs. Finally, OT staff perceived any materials that touched the patient as contaminated and discarded them in the contaminated waste bin. On one list, 25 uncontaminated pairs of NSGs were discarded in the infectious (orange) bin out of fear of transmitting infection (Beliefs about consequences)—the theater manager said, “It makes sense for me to throw all gloves in the orange bin; what if they’re contaminated?” (O6; N1).

#### Theme 4: Striving for “Efficiency”

Another influential factor driving practices in OTs is the striving to finish the operation list as quickly as possible (Goals). OT staff’s striving to finish an operation quickly drives wasteful practices. For example, unnecessarily opening extra swab packs, leaving monitors and computers on, and donning NSGs to be ready in case they need to perform a task, were time-saving unsustainable behaviors which were commonly observed. Even ingrained habitual behaviors such as donning NSGs are momentarily set aside as “efficiency” takes precedence. For example, a nurse unnecessarily grabbed a pair of NSGs with the intention to don them to move a patient. As the team wanted to perform the task quickly, the nurse opted to perform the task without NSGs (O3; N5).

#### Theme 5: Sustainability Practices are Influenced by Leadership and Communication Between Staff

Leadership (management and senior staff members) influenced sustainability through organizational policy, and through communication during the briefing. When senior surgeons led the brief and mentioned sustainability (which materials and instruments to open, and which to have on stand-by), less waste was produced (Social influences). In contrast, SpR-led briefings focused on the clinical aspects and did not cover sustainability, resulting in more waste. This finding is based on overall counts of consumables (swabs and sutures) in comparable operations, reflecting patterns in communication influencing waste generation. Communicating the items needed for the operation by the surgeons to the nurses also reduced waste (Knowledge). A theater manager explained that the best way to drive sustainable practices is by changing the Trust’s policy—“We do what we are told, they need to change policy so that we change what we do; if they tell us, we will do it” (Environmental context and resources).

#### Theme 6: Unsustainable Practices are Exacerbated by the Physical Environment in OTs

**FIGURE 4.**
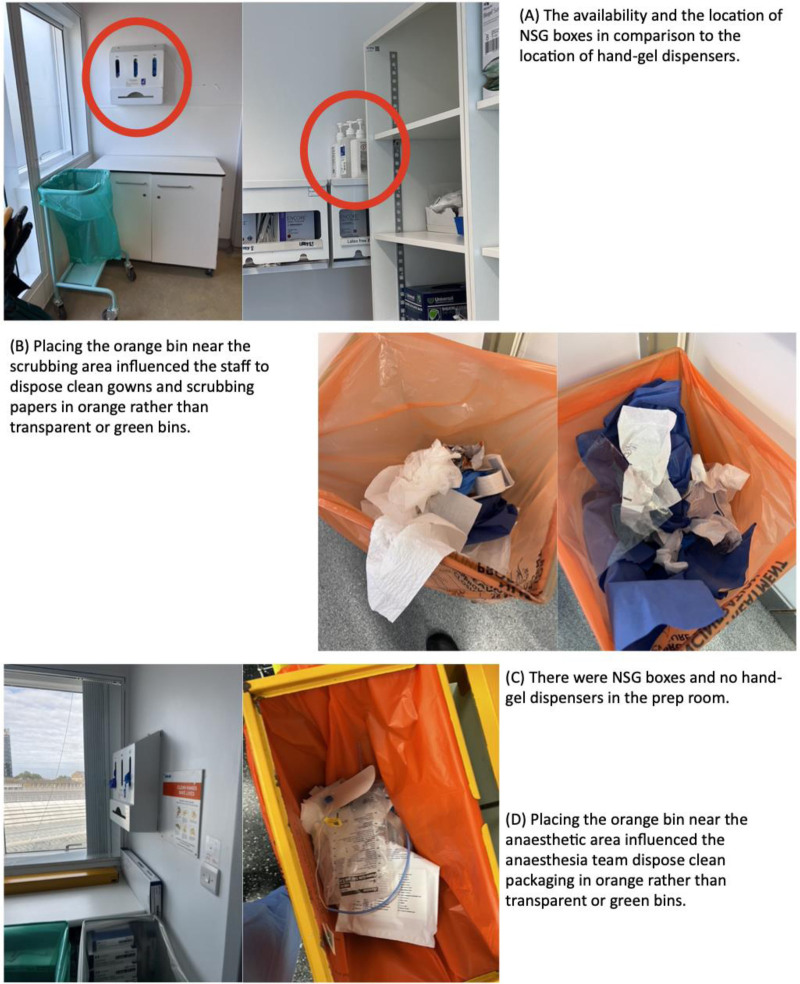
Photographic images demonstrating that OT setup encourages unsustainable practices. Photograph (A) shows 2 hand gel dispensers hidden behind a cupboard in the scrub room, making them less accessible than the NSG dispenser placed at the entrance. Photograph (B) highlights the placement of infectious contaminated waste (orange) bins near the scrub area, leading staff to mistakenly discard clean gown and scrub papers into the orange bin instead of the clear, mixed-recycling bin. Photograph (C) illustrates the absence of gel dispensers and the prominence of the NSG dispenser in the prep room, which may prompt staff to use NSGs instead of hand gel. Photograph (D) again demonstrates how placing the orange bins near the anesthesia area encourages the anesthetist and anesthesia nurse to incorrectly dispose of clean packaging in the orange bins, instead of the appropriate clear, mixed-recycling bin.

OT setup led to energy waste, encouraged the use of NSGs, and the disposal of waste in orange bins. Given that sustainability is not an established driver of practices, the physical environment can substantially affect staff behaviors (Environmental context and resources) (Fig. 4). For example, placing infectious contaminated-waste bins near the scrub area exacerbates incorrect waste disposal, as ease facilitates disposing clean items in these bins. Furthermore, the abundance of NSG boxes in comparison to the relative inaccessibility of gel dispensers contributes to their overuse.

## DISCUSSION

To our knowledge, this is the first ethnographic study investigating the context and drivers behind sustainability practices in OTs, providing invaluable insights for policymakers. The most common unsustainable behaviors were incorrect NSG overuse (potentially compromising safety), and incorrect waste segregation and disposal. Unsustainable practices were found to be consistent, repetitive, habitual, and ritual-like,^43^ in contrast to sustainable practices that were infrequent and easily abandoned, indicating a weak sustainability culture. Thematic analysis also revealed that unsustainable practices start during early training, driven by speed and unevidenced precaution, and are influenced by leadership and the physical environment in OTs.

In our institution, elective patients undergo preoperative screening for blood-borne infections and resistant organisms, and current guidelines and our policy state that NSGs should only be used when coming in contact with blood and bodily fluids.^44–46^ Nevertheless, the excessive fear of infection led to disproportionate and unevidenced precautionary practices (Emotions). Operating staff overestimated the risk of infection, resulting in the unnecessary use of protective equipment for low-risk tasks and incorrect disposal. Uncontaminated materials that came into contact with patients were discarded as contaminated waste. These practices are compounded by emotional responses, which seem to relate to disgust, leading to the use of NSGs for tasks in which they are not required. This is consistent with the findings of previous studies, which concluded that healthcare professionals’ decisions about NSG use are primarily influenced by emotions like fear and disgust, along with the norms and practices established within their profession and organization.^47,48^ Interventions should address these influences through evidence-based education and behavioral modeling through sustainability champions.^12^

A study published before the COVID-19 pandemic reported that 6–7 pairs of NSGs were used inappropriately per operation.^49^ In contrast, the current study demonstrated a higher average of 8–10 pairs per procedure, reflecting a culture of excessive NSG use, driven by ingrained habits and institutional norms rather than evidence. Current guidelines state that alcohol-based sanitizer should be used before and after coming in contact with the patient or performing a procedure that requires NSG.^50^ As such, the ratio of hand gel to NSG use should be >1. In our study, the staff chose to unnecessarily don NSGs when coming in contact with patients, instead of sanitizing their hands. As such, this ratio was less than 0.3, indicating that unnecessary overuse often comes at the expense of appropriate hand sanitization.^51–53^ Other studies also demonstrated that the overuse of NSGs leads to inappropriate use and poor hand hygiene, which compromises safety by increasing transmission and cross-contamination of infection, putting both patients and staff at risk of healthcare-associated infections.^48,54^ This was the most consistent and evident example where unsustainable practices compromised patient and staff safety by directly contravening infection prevention guidance. We note that while other practices (eg, the incorrect segregation of waste, the precautionary unevidenced use of antibiotics, or catheterization)^55^ could also present risks to patients, these were not classified as unsustainable, because they fell within the scope of clinical decision-making without binary policies against which they could be compared.

Temporal factors (events related to time) play a vital role in shaping behaviors.^56,57^ Unsustainable practices, such as double-gloving, start during early clinical training, where individuals observe and emulate behaviors from peers and seniors.^58^ As these practices are commonly repeated in the consistent context of the OT, and are reinforced by influences such as fear of contracting infections, these practices become habitual and are performed automatically.^59,60^ Past nontraining experiences also play a role in shaping staff’s attitudes and behaviors. This is exemplified in the heightened risk-aversion behaviors observed during the COVID-19 pandemic.^61,62^ These combinations lead to the perpetuation of practices that are wasteful and unevidenced.

In this study, nurses were more inclined to adopt unsustainable practices, which may be influenced by the perceived expectations associated with their role (TDF: Social/professional role and identity). Nurses exhibited higher rates of unsustainable practices, particularly excessive NSG use, compared to surgeons. This disparity suggests that professional identity plays a critical role in shaping sustainability behaviors in OTs. Nurses often adopt a more risk-averse approach, reinforcing unsustainable practices such as habitual NSG overuse and incorrect waste disposal.^63^ In contrast, surgeons, who focus primarily on procedural efficiency and clinical outcomes, may exhibit less precautionary-driven behavior. Research shows variation in the attitudes of different healthcare staff towards health guidelines and rules, largely due to their perception of the safety culture, which may lead to inconsistent practices.^64^ For example, McDonald et al^64^ reported higher rates of NSGs overuse among nurses compared with surgeons. To improve sustainability in OTs, interventions should acknowledge and target these role-based differences. Tailored educational programs should address nurses’ heightened risk perceptions and integrate sustainability principles into infection prevention training. Additionally, leveraging leadership within nursing and surgical teams to model sustainable practices could facilitate behavior change, embedding sustainability into professional identity.

Behavioral studies demonstrate that individuals often engage in practices they perceive as beneficial in the short term, even if they entail potential negative long-term consequences.^65–67^ Under time pressure, OT staff often prioritize completing operations quickly over adhering to sustainable practices (TDF: Goals). This striving for efficiency manifests in behaviors such as opening swab packs preemptively, leaving monitors and computers running, and donning NSGs just in case they needed to perform a task–a documented behavior in healthcare literature.^14,68^ Importantly, our findings distinguish between efficiency, which can reduce operative time, anesthetic exposure, and downstream resource use, and the striving for efficiency, which drives behaviors that are motivated by trying to save time (without necessarily having this effect) and are likely ultimately wasteful.^13^ The current study demonstrated that speed-driven practices can override habitual unsustainable precautionary practices, which can be described as theater rituals^43^ (standardized, rule-bound, predictable, and repetitive), highlighting the strength of the influence of “striving for ‘efficiency’” in the context of OTs.

Addressing sustainability in OTs requires a culture change at the organizational level, with leadership prioritizing sustainability through systemic policy changes. When individual departments such as infection prevention and control, and domestic and waste management make decisions based on their own competing priorities, changing practice at the level of individual employees becomes challenging. While observation of behaviors and devising behavioral interventions at the staff level is an essential step, achieving sustainable change may require policy change, such as procurement policies, infrastructure modifications, and regulatory enforcement.^69^ Leadership can drive change by embedding sustainability targets into institutional policies, mandating training, and incentivising compliance.^70^ Financial and logistical barriers, including upfront costs and workflow disruptions, may hinder adoption; however, strategic investments in reusable surgical instruments, biodegradable materials, and circular economy principles can reduce long-term costs and waste.^4^ As such, achieving sustainability in OTs requires a dual approach, integrating top-down policy enforcement with bottom-up behavioral change to embed sustainability into routine clinical practice.

While improving sustainability often requires senior-level input (eg, leadership and management), which was not included in the present study, the observations highlight key areas that may be most productive to address. For example, establishing clear institutional policies on PPE use, such as when to double-glove or wear eye-protection, would reduce ambiguity and align behaviors with evidence-based guidance. Improving labeling and signage in OTs, including waste bins, would reduce unnecessary waste, and would support sustainability goals and offer significant financial benefits. According to NHS England’s Clinical Waste Strategy Review, optimizing waste segregation can reduce high-temperature incineration requirements by 35% and alternative treatment infrastructure needs by 61%.^71^ According to Estates Return Information Collection figures 2020/21, such improvements could save the NHS over £11 million annually (15% of total clinical and offensive waste expenditure) and cut whole-life carbon emissions by 29,540 tonnes per year, equivalent to a 30% reduction in CO₂ emissions from NHS waste management.^71^

Nontechnical skills such as communication and shared knowledge are needed to ensure sustainable changes in complex clinical situations.^72^ Specifically, staff may discuss surgical plans and establish a shared mental model of the sustainable practices to be performed during surgery.^73,74^ The Intercollegiate Green Theater Checklist can facilitate this collaborative approach by coordinating efforts to minimize waste and optimize resource use.^55^ By fostering effective communication, the team can ensure that sustainability goals are integrated into their practice, ultimately contributing to more sustainable, efficient, and safe surgical procedures. Additionally, supply chain reforms, such as introducing reusable instruments and biodegradable materials can reinforce behavior change by making sustainable choices easier and more accessible. The James Lind Alliance Priority Setting Partnership designated the “sustainable reusable equipment” and the “procurement of medicines, equipment and items” as the top 2 priorities for enabling greener OTs.^16^ Aligning procurement with circular economy principles not only reduces emissions but can support clinical staff in adopting environmentally responsible practices, creating a coherent framework for sustainable surgical care.

### Study Limitations

While this study was conducted in a specific geographical setting and focused on elective surgical cases (potentially limiting generalisability to emergency contexts) the in-depth insights generated are intended to be transferable rather than broadly generalizable.^75^ Transferability allows researchers and practitioners to assess whether study insights are applicable to their own settings by considering the relevance of identified themes and influences.^76,77^ Furthermore, many of the behavioral drivers observed in our study align with existing healthcare literature, suggesting that similar behavioral patterns are present in other healthcare settings.^78^

The study relied on observational data, which may have been subject to observer bias, particularly in areas lacking formal institutional policies, such as in relation to double-gloving and eye-protection, which could not be standardized. Additionally, although an inherent limitation of ethnography, we attempted to mitigate the potential impact of the Hawthorne effect. To become embedded in the OT environment, the researchers donned theater attire and positioned themselves in a way that did not disturb the surgical procedure. Additionally, the staff were not informed about the specific details of what was being observed, with only the theater manager being made aware, in order to limit any influence on their behaviors.

Surgeons’ choices of operative equipment and approach, which are important for sustainability, were noted. However, equipment accounts for a significantly smaller proportion of OT emissions (5%) compared with consumables (32%), and decisions are often influenced by procurement policies and clinical imperatives.^79^ As such, we concluded that these issues would be better explored in a qualitative interview study in which the nuances of decision-making can be explored in the context of the type of operation, the list of operations to be performed, and the organization.

Nevertheless, the strength of this study lies in the real-time observation of sustainability barriers and behavioral choices in the OT context. The study leveraged the reflective insights of the authors, employing implicit, reflective analysis throughout the research process to ensure rigour, trustworthiness, and transferability of the findings.^80–83^ Furthermore, the use of the TDF yielded a more nuanced analysis, and fits within a wider process for understanding and changing behavior, including the Theory and Techniques Tool,^84^ and the Behavior Change Techniques Taxonomy,^23^ which can be used to inform the design of future behavioral interventions. As such, these insights should be utilized for designing sustainability interventions in OTs to safely achieve sustainability and net-zero targets.

Further research employing nonobservational methods, such as questionnaires, interviews, and behavioral frameworks, is needed to explore nonobservable influences, such as individual contextual experiences, to enhance our understanding of sustainability practices in OTs. By highlighting the inconsistency of sustainable practices in OTs and identifying the nuanced drivers of unsustainable behaviors, this study can be used to inform the design of more effective interventions, that can be feasibly incorporated into the OT setting. Given the multiple drivers of unsustainable practices, multifaceted interventions may be needed to promote more sustainable practices.^12^ In addition to making changes to the physical environment of OTs to facilitate more sustainable practices, such interventions should aim to address OT staff’s perceptions and attitudes towards sustainability. This can be achieved through continuous education and training on the impact of unsustainable practices. Additionally, they should address the powerful unconscious influences at play (eg, through changes to the OT context) and model positive behavior by capitalizing on the influence of leadership and sustainability champions.

## CONCLUSIONS

This study revealed the prevalence of unsustainable practices in OTs, which may compromise patient safety. It also provides a nuanced understanding of unsustainable practices and their drivers in OTs using the TDF, which can be used to inform the design of impactful behavioral interventions. Multiple drivers influence unsustainable practices, including the physical environment, professional role, ingrained habits, the striving for efficiency, and unevidenced precaution, which may require a multifaceted intervention to promote more sustainable practices. For example, behavioral modeling by nurses in the role of sustainability champions, changes to the physical environment of OTs to facilitate more sustainable practices, and educating staff about infection risks and the sustainability implications of different practices.

## Acknowledgments

All authors contributed to manuscript revision, read, and approved the submitted version. The Guarantor confirms that all contributing authors approved the final manuscript.

## Supplementary Material

## References

[1] Anthony C Abbas M Allen A. Managing the health effects of climate change: lancet and University College London Institute for Global Health Commission. Lancet. 2009;373:1693–1733. 19447250 10.1016/S0140-6736(09)60935-1

[2] Greener NHS. Delivering a ‘Net Zero’ National Health Service. Greener NHS. Available at: https://www.england.nhs.uk/greenernhs/publication/delivering-a-net-zero-national-health-service/. Accessed February 26, 2024.

[3] MacNeill AJ Lillywhite R Brown CJ. The impact of surgery on global climate: a carbon footprinting study of operating theatres in three health systems. Lancet Planet Health. 2017;1:e381–e388. 29851650 10.1016/S2542-5196(17)30162-6

[4] Rizan C Steinbach I Nicholson R. The carbon footprint of surgical operations: a systematic review. Ann Surg. 2020;272:986–995. 32516230 10.1097/SLA.0000000000003951

[5] Thiel CL Eckelman M Guido R. Environmental impacts of surgical procedures: life cycle assessment of hysterectomy in the United States. Environ Sci Technol. 2015;49:1779–1786. 25517602 10.1021/es504719g PMC4319686

[6] Bravo D Townsend CB Tulipan J. Economic and environmental impacts of the wide-awake, local anesthesia, no tourniquet (WALANT) technique in hand surgery: a review of the literature. J Hand Surg Glob Online. 2022;4:456–463. 36425376 10.1016/j.jhsg.2022.05.009 PMC9678698

[7] Drew J Christie SD Tyedmers P. Operating in a climate crisis: a state-of-the-science review of life cycle assessment within surgical and anesthetic care. Environ Health Perspect. 2021;129:76001. 34251875 10.1289/EHP8666 PMC8274692

[8] Kwakye G Brat GA Makary MA. Green surgical practices for health care. Arch Surg. 2011;146:131–136. 21339421 10.1001/archsurg.2010.343

[9] Perry H Reeves N Ansell J. Innovations towards achieving environmentally sustainable operating theatres: a systematic review. Surgeon. 2023;21:141–151. 35715311 10.1016/j.surge.2022.04.012

[10] Southorn T Norrish A Gardner K. Reducing the carbon footprint of the operating theatre: a multicentre quality improvement report. J Perioper Pract. 2013;23:144–146. 23909168 10.1177/175045891302300605

[11] Thiel CL Woods NC Bilec MM. Strategies to reduce greenhouse gas emissions from laparoscopic surgery. Am J Public Health. 2018;108:S158–S164. 29698098 10.2105/AJPH.2018.304397 PMC5922216

[12] Almukhtar A Batcup C Bowman M. Interventions to achieve environmentally sustainable operating theatres: an umbrella systematic review using the behaviour change wheel. Int J Surg. 2024;110:7245–7267. 39093843 10.1097/JS9.0000000000001951 PMC11573083

[13] Robb HD Pegna V. The intercollegiate green theatre checklist. RCS bulletin. 2023;105:64–67.

[14] Almukhtar A Batcup C Bowman M. Barriers and facilitators to sustainable operating theatres: a systematic review using the Theoretical Domains Framework. Int J Surg. 2024;110:554–568. 37889570 10.1097/JS9.0000000000000829 PMC10793789

[15] Lodhia S Pegna V Abrams R. Improving environmental sustainability of operating theatres: a systematic review of staff attitudes, barriers, and enablers. Ann Surg. 2024;280:954–959. 38726670 10.1097/SLA.0000000000006337

[16] Clayton-Smith M Narayanan H Shelton C. Greener operations: a James Lind alliance priority setting partnership to define research priorities in environmentally sustainable perioperative practice through a structured consensus approach. BMJ Open. 2023;13:e066622. 10.1136/bmjopen-2022-066622 PMC10069275 36977540

[17] Braaf S Manias E Riley R. The ‘time-out’ procedure: an institutional ethnography of how it is conducted in actual clinical practice. BMJ Qual Saf. 2013;22:647–655. 10.1136/bmjqs-2012-001702 23584209

[18] Ziman R Espin S Grant RE. Looking beyond the checklist: an ethnography of interprofessional operating room safety cultures. J Interprof Care. 2018;32:575–583. 29630424 10.1080/13561820.2018.1459514

[19] Rydenfält C. Safety-II and the study of healthcare safety routines: two paths forward for research. J Patient Saf Risk Manag. 2022;27:124–128.

[20] van Harten A Gooszen HG Koksma JJ. An observational study of distractions in the operating theatre. Anaesthesia. 2021;76:346–356. 33252139 10.1111/anae.15217 PMC7891421

[21] Atkins L Francis J Islam R. A guide to using the theoretical domains framework of behaviour change to investigate implementation problems. Implement Sci. 2017;12:77. 28637486 10.1186/s13012-017-0605-9 PMC5480145

[22] Cane J O’Connor D Michie S. Validation of the theoretical domains framework for use in behaviour change and implementation research. Implement Sci. 2012;7:37. 22530986 10.1186/1748-5908-7-37 PMC3483008

[23] Marques MM Wright AJ Corker E. The behaviour change technique ontology: transforming the behaviour change technique taxonomy v1. Wellcome Open Res. 2023;8:308. 37593567 10.12688/wellcomeopenres.19363.2 PMC10427801

[24] Michie S Carey RN Johnston M. From theory-inspired to theory-based interventions: a protocol for developing and testing a methodology for linking behaviour change techniques to theoretical mechanisms of action. Ann Behav Med. 2017;52:501–512. 10.1007/s12160-016-9816-6 PMC6367898 27401001

[25] Marks A O’Mahoney J. Researching identity: a critical realist approach’. in Edwards PK O’Mahoney J Steve Vincent S(eds), Studying Organizations Using Critical Realism: A Practical Guide. Oxford University Press; 2014.

[26] Allen D. Ethnomethodological insights into insider–outsider relationships in nursing ethnographies of healthcare settings. Nurs Inq. 2004;11:14–24. 14962343 10.1111/j.1440-1800.2004.00201.x

[27] Aude F Jean-Louis B Catherine G. Barriers to staff adoption of a surgical safety checklist. BMJ Qual Saf. 2012;21:191. 10.1136/bmjqs-2011-000094 PMC3285141 22069112

[28] Yuan CT Walsh D Tomarken JL. Incorporating the World Health Organization surgical safety checklist into practice at two hospitals in Liberia. Jt Comm J Qual Patient Saf. 2012;38:254–260. 22737776 10.1016/s1553-7250(12)38032-x

[29] Malterud K Siersma VD Guassora AD. Sample size in qualitative interview studies: guided by information power. Qual Health Res. 2016;26:1753–1760. 26613970 10.1177/1049732315617444

[30] Hennink MM Kaiser BN Marconi VC. Code saturation versus meaning saturation: how many interviews are enough? Qual Health Res. 2017;27:591–608. 27670770 10.1177/1049732316665344 PMC9359070

[31] Fusch P Ness LR. Are we there yet? Data saturation in qualitative research. Qual Rep. 2015;20:1408–1416.

[32] Tjørnhøj-Thomsen T Hansen HP. Ethnographic Fieldwork. In: Facey KM Ploug Hansen H Single ANV, eds. Patient Involvement in Health Technology Assessment. Springer; 2017:149–163.

[33] Whitehead TL. Basic Classical Ethnographic Research Methods Secondary Data Analysis, Fieldwork, Observation/Participant Observation, and Informal and Semi-Structured Interviewing. 2005. Available at: https://static1.squarespace.com/static/542d69f6e4b0a8f6e9b48384/t/573b7ebdc2ea515a3fd6b4c2/1463516862124/Classical-Basic+Ethnographic+Methods.pdf. Accessed December 7, 2025.

[34] Kitto SC Chesters J Grbich C. Quality in qualitative research. Med J Aust. 2008;188:243–246. 18279135 10.5694/j.1326-5377.2008.tb01595.x

[35] St Germaine RL Hanson J de Gara CJ. Double gloving and practice attitudes among surgeons. Am J Surg. 2003;185:141–145. 12559444 10.1016/s0002-9610(02)01217-5

[36] Makama JG Okeme IM Makama EJ. Glove perforation rate in surgery: a randomized, controlled study to evaluate the efficacy of double gloving. Surg Infect. 2016;17:436–442. 10.1089/sur.2015.165 26981792

[37] Roebuck A Harrison EM. Operating theatre etiquette, sterile technique and surgical site preparation. Surgery (Oxford). 2017;35:177–184.

[38] Laine T Aarnio P. How often does glove perforation occur in surgery? comparison between single gloves and a double-gloving system. Am J Surg. 2001;181:564–566. 11513787 10.1016/s0002-9610(01)00626-2

[39] Bingham AJ Witkowsky P. Deductive and inductive approaches to qualitative data analysis. Analyzing and Interpreting Qualitative Data: After the Interview. 2021;1:133–146.

[40] Braun V Clarke V. Using thematic analysis in psychology. Qual Res Psychol. 2006;3:77–101.

[41] Fereday J Muir-Cochrane E. Demonstrating rigor using thematic analysis: a hybrid approach of inductive and deductive coding and theme development. Int J Qual Methods. 2006;5:80–92.

[42] Tong A Sainsbury P Craig J. Consolidated criteria for reporting qualitative research (COREQ): a 32-item checklist for interviews and focus groups. Int J Qual Health Care. 2007;19:349–357. 17872937 10.1093/intqhc/mzm042

[43] Smith ACT Stewart B. Organizational rituals: features, functions and mechanisms. Int J Manag Rev. 2011;13:113–133.

[44] Lynch P Jackson MM Cummings MJ. Rethinking the role of isolation practices in the prevention of nosocomial infections. Ann Intern Med. 1987;107:243–246. 3605901 10.7326/0003-4819-107-2-243

[45] Pratt R Pellowe C Wilson J. epic2: national evidence-based guidelines for preventing healthcare-associated infections in NHS hospitals in England. J Hosp Infect. 2007;65:S1–S59. 17307562 10.1016/S0195-6701(07)60002-4 PMC7134414

[46] Wilson J Breedon P. Universal precautions. Nurs Times. 1990;86:67–70. 2399158

[47] Loveday HP Lynam S Singleton J. Clinical glove use: healthcare workers’ actions and perceptions. J Hosp Infect. 2014;86:110–116. 24412643 10.1016/j.jhin.2013.11.003

[48] Wilson J Bak A Loveday HP. Applying human factors and ergonomics to the misuse of nonsterile clinical gloves in acute care. Am J Infect Control. 2017;45:779–786. 28365143 10.1016/j.ajic.2017.02.019

[49] Krediet AC Kalkman CJ Bonten MJ. Hand-hygiene practices in the operating theatre: an observational study. Br J Anaesth. 2011;107:553–558. 21665900 10.1093/bja/aer162

[50] World Health Organization. WHO Guidelines on Hand Hygiene in Health Care. 2009. 10 February 2025. Available at: https://www.who.int/publications/i/item/9789241597906. Accessed February 10, 2025.

[51] Flores A Pevalin DJ. Healthcare workers’ compliance with glove use and the effect of glove use on hand hygiene compliance. Bri J Infect Control. 2006;7:15–19.

[52] Fuller C Savage J Besser S. “The dirty hand in the latex glove”: a study of hand hygiene compliance when gloves are worn. Infect Control Hosp Epidemiol. 2011;32:1194–1199. 22080658 10.1086/662619

[53] World Health Organization. Hand hygiene technical reference manual: to be used by health-care workers, trainers and observers of hand hygiene practices. World Health Organization. Available at: https://www.who.int/publications/i/item/9789241598606. Accessed February 10, 2025.

[54] Girou E Chai SHT Oppein F. Misuse of gloves: the foundation for poor compliance with hand hygiene and potential for microbial transmission? J Hosp Infect. 2004;57:162–169. 15183248 10.1016/j.jhin.2004.03.010

[55] Winter Beatty J Douglas Robb H Chu J Pegna V Tresta F Hurst K. Intercollegiate Green Theatre Checklist V0.2: Compendium of Evidence. Royal College of Surgeons of Edinburgh, Royal College of Physicians and Surgeons of Glasgow, Royal College of Surgeons of Ireland, Royal College of Surgeons of England. 2025. Available at: https://www.rcsed.ac.uk/media/vspi2d5g/green-theatre-checklist-compendium-of-evidence-document.pdf. Accessed March 14, 2025.

[56] Liberman N Trope Y. Construal Level Theory of Intertemporal Judgment and Decision in Loewenstein, Read & Baumeister’s Time and Decision: Economic and Psychological Perspectives of Intertemporal Choice. Russell Sage Foundation; 2003:chap 245–277.

[57] Loewenstein G Thaler RH. Anomalies: intertemporal choice. J Econ Perspect. 1989;3:181–193.

[58] Hearld LR Bleser WK Alexander JA. A systematic review of the literature on the sustainability of community health collaboratives. Med Care Res Rev. 2016;73:127–181. 26429834 10.1177/1077558715607162

[59] Orbell S Verplanken B. The automatic component of habit in health behavior: habit as cue-contingent automaticity. Health Psychol. 2010;29:374–383. 20658824 10.1037/a0019596

[60] Wood W Neal DT. A new look at habits and the habit-goal interface. Psychol Rev. 2007;114:843–863. 17907866 10.1037/0033-295X.114.4.843

[61] Mekonnen BA Aragaw TA. Environmental Sustainability and COVID-19 pandemic: an overview review on new opportunities and challenges. In: Muthu SS, ed. COVID-19: Environmental Sustainability and Sustainable Development Goals. Springer; 2021:117–140.

[62] Mondal R Mishra S Pillai JSK. COVID 19 pandemic and biomedical waste management practices in healthcare system. J Family Med Prim Care. 2022;11:439–446. 35360761 10.4103/jfmpc.jfmpc_1139_21 PMC8963639

[63] Stein AD Makarawo TP Ahmad MFR. A survey of doctors’ and nurses’ knowledge, attitudes and compliance with infection control guidelines in Birmingham teaching hospitals. J Hosp Infect. 2003;54:68–73. 12767850 10.1016/s0195-6701(03)00074-4

[64] McDonald R Waring J Harrison S. Rules and guidelines in clinical practice: a qualitative study in operating theatres of doctors’ and nurses’ views. Qual Saf Health Care. 2005;14:290–294. 16076795 10.1136/qshc.2005.013912 PMC1744048

[65] Hall GTF Peter A. Reducing Adolescent Risk: Toward an Integrated Approach. SAGE Publications, Inc.; 2003:106:chap 13.

[66] Strathman A Gleicher F Boninger DS. The consideration of future consequences: weighing immediate and distant outcomes of behavior. J Pers Soc Psychol. 1994;66:742–752.

[67] Zimbardo PG Boyd JN. Putting time in perspective: a valid, reliable individual-differences metric. In: Stolarski M Fieulaine N van Beek W, eds. Time Perspective Theory; Review, Research and Application: Essays in Honor of Philip G Zimbardo. Springer International Publishing; 2015:17–55.

[68] Sürme Y Maraş G. Recycling, responsible consumption and nursing: a qualitative study of surgical nurses’ recycling and medical waste management. J Nurs Manag. 2022;30:4514–4522. 36326215 10.1111/jonm.13891

[69] McGain F Muret J Lawson C. Environmental sustainability in anaesthesia and critical care. Br J Anaesth. 2020;125:680–692. 32798068 10.1016/j.bja.2020.06.055 PMC7421303

[70] Sherman JD Thiel C MacNeill A. The green print: advancement of environmental sustainability in healthcare. Resour Conserv Recycl. 2020;161:104882.

[71] NHS England. NHS Clinical Waste Strategy. NHS Engalnd. Updated 7 March 2023. 2025. Available at: https://www.england.nhs.uk/long-read/nhs-clinical-waste-strategy/. Accessed February 10, 2025.

[72] Gittell JH Fairfield KM Bierbaum B. Impact of relational coordination on quality of care, postoperative pain and functioning, and length of stay: a nine-hospital study of surgical patients. Med Care. 2000;38:807–819. 10929993 10.1097/00005650-200008000-00005

[73] Bogdanovic J Perry J Guggenheim M. Adaptive coordination in surgical teams: an interview study. BMC Health Serv Res. 2015;15:128. 25889397 10.1186/s12913-015-0792-5 PMC4389413

[74] Burtscher MJ Manser T. Team mental models and their potential to improve teamwork and safety: a review and implications for future research in healthcare. Saf Sci. 2012;50:1344–1354.

[75] Lincoln YS Guba EG. Naturalistic Inquiry. Sage; 1985.

[76] Drisko JW. Transferability and generalization in qualitative research. Res Soc Work Pract. 2025;35:102–110.

[77] Polit DF Beck CT. Generalization in quantitative and qualitative research: myths and strategies. Int J Nurs Stud. 2010;47:1451–1458. 20598692 10.1016/j.ijnurstu.2010.06.004

[78] Dixon-Woods M McNicol S Martin G. Ten challenges in improving quality in healthcare: lessons from the Health Foundation’s programme evaluations and relevant literature. BMJ Qual Saf. 2012;21:876–884. 10.1136/bmjqs-2011-000760 PMC3461644 22543475

[79] Whiting A Tennison I Roschnik S. Surgery and the NHS Carbon Footprint. RCS bulletin. 2020;102:182–185.

[80] Finlay L. “Outing” the researcher: the provenance, process, and practice of reflexivity. Qual Health Res. 2002;12:531–545. 11939252 10.1177/104973202129120052

[81] Gough B. Deconstructing Reflexivity. In Reflexivity. In: Finlay L Gough B, eds. Reflexivity: A Practical Guide for Researchers in Health and Social Sciences. 2003:3–20.

[82] Pillow W. Confession, catharsis, or cure? Rethinking the uses of reflexivity as methodological power in qualitative research. Int J Qual Stud Educ. 2003;16:175–196.

[83] Smith JA. Towards reflexive practice: engaging participants as co-researchers or co-analysts in psychological inquiry. J Community Appl Soc Psychol. 1994;4:253–260.

[84] Johnston M Carey RN Connell Bohlen LE. Development of an online tool for linking behavior change techniques and mechanisms of action based on triangulation of findings from literature synthesis and expert consensus. Transl. Behav. Med. 2020;11:1049–1065. 10.1093/tbm/ibaa050 PMC8158171 32749460

