# Supplementary material for: Understanding Sustainability in Operating Theaters: An Ethnographic Study to Determine Drivers of Unsustainable Behaviors
Source: Ann Surg Open. 2026 Jan 5;7(1):e635. doi: 10.1097/AS9.0000000000000635 (PMC13016191; doi:10.1097/AS9.0000000000000635)
Supplement: Supplementary file 1 [file as9-7-e635-s001.pdf]

Supplementary Digital Content

Table 1: Shortened version of the observation template used to guide data collection

Researcher's Information:

Observation Date:

Observations Location:

Observation Start Time: Finish Time:

Speciality:
Emergency/Elective?

Operation(s) Type of anaesthesia: Can it be done under Regional/Local?
Why/Why not?

Name of Theatre Coordinator:

Signature for Consent:

Staff:

Roles (grouped)	How many	Gloves/details	Caps/details

Environment:

Item	Details – theatre/prep room/ anaesthesia room

**Observation Points**	**Contextual Details / stage of operation/ Notes**		
glove is used unnecessarily	Preop		
	Intraop		
	Postop		
Time/Place	*Who/What*	*Behaviour*	*Reflections*

**Observation Points**	**Contextual Details / stage of operation / Notes for critical case**		
Time/Place	*Who/What*	*Behaviour*	*Reflections*

Table 2: Staff group and numbers, and speciality of observed procedures and the number of hours

Speciality	Number of hours observed
General surgery	14.25
Colorectal surgery	12.75
Breast surgery	5.5
Bariatrics surgery	5.5
Chronic pain	4

Staff group	Combined number
Theatre nurse	27
Higher Speciality Surgical Registrar (SpR)	7
Anaesthesia consultant	7
Surgical consultants	6
Anaesthesia nurse	4
Senior House Officers (SHO) – surgery	3
Anaesthesia trainee	3
Operating Theatre Practitioners (ODP)	3
Medical student	2
Radiographer - senior	1
Radiographer - junior	1

Table 3: Evidence, Guidance, and Policies

Practice/Topic	Guidance/Policy Summary
**Personal Protective Equipment (PPE) practices: wearing non-sterile gloves**<br><br>Current guidelines state that NSGs should be used when coming in contact with blood, bodily fluids, or intact mucus membranes.[1-3] This is reflected in our Trust and the wider National Health Service (NHS) policy.	**Gloves are to be worn only when indicated**<br><br>i. Exposure to blood and/or other body fluids including excreta, non-intact skin or mucous membranes (National Health Service 2023; Hand Hygiene Policy V3.1, 2024)<br><br>ii. Aseptic/surgical (9) and invasive procedures (Girou et al, 2004)<br><br>iii. Handling sharps or contaminated devices procedures (Girou et al, 2004)<br><br>iv. Coming into contact with visibly contaminated items and surfaces (WHO, 2010)<br><br>v. As indicated for the isolation of patients (see trust isolation policy) (Hand Hygiene Policy V3.1, 2024)<br><br>If gloves are worn to perform a procedure, hands must be cleaned prior to putting on the gloves and they must be removed immediately after the procedure and hands cleaned as indicated in the 5 moments for hand hygiene (Hand Hygiene Policy V3.1, 2024)

**Personal Protective Equipment (PPE) practices: wearing additional sterile gloves (double-gloving) and the use of eye-protection (face-shield)):**	There is no formal policy on double gloving and the routine use of eye-protection, and these are not standardised practices in our institution, in common with many others around the world.[4] When this study was conducted, evidence for and against double-gloving in operating theatres was equivocal.[5,6] A Cochrane meta-analysis demonstrated no direct evidence that double-gloving reduced surgical site infections.[7] The use of indicator gloves (gloves with colour contrast) has been proposed as an additional safeguard, facilitating intraoperative detection of glove perforations, and therefore protection against blood-born infections; however, the evidence to support this is moderate.[8] Prospective randomised studies demonstrated that higher numbers of perforations occurred during emergency surgery, at night time, or when the primary surgeon was a registrar (trainee)[9,10], and double gloving is more commonly practiced in specialties where sharp structures are encountered, such as Orthopaedics and Dentistry.[6]<br><br>Similarly, the use of surgical masks with eye-protection is intended to protect the surgical staff from splatter. Current guidelines recommend eye protection only when splash or spray of blood or body fluids is anticipated, and not for all surgeries.[11-13] Therefore, they are more commonly used in high-risk procedures such as orthopaedics, trauma, and major vascular surgery. For the purpose of these behaviours, these were considered high-risk procedures. The risk in laparoscopic surgery is considerably lower than

	that in open surgery, and therefore was not considered a high-risk procedure.[14]<br><br>In our institution and many institutions around the world, all elective patients undergo pre-operative screening and are considered high risk if found to have a blood-born infection or are carriers of a resistant organism. These were also considered high-risk procedures.<br><br>As such, double gloving and the use of eye protection were not categorised as universally sustainable or unsustainable. Instead, they were examined as contrasting practices to explore the underlying behavioural drivers which led staff to choose one over the other in low-risk contexts. Furthermore, instances where the staff opted to double-glove were discussed with the participants to identify the drivers, and then within the research team. Where there was doubt about whether double-gloving or other protective measures were clinically indicated, these were not considered unsustainable. This approach allowed us to interpret behaviours within their clinical and institutional context and to identify the drivers behind them (e.g. fear of contracting infections, and automatic behaviours (habits)).
**Waste disposal and processing: Definition and management of clinical waste[15]**	**Clinical waste is:**<br><br>I. Waste containing viable micro-organisms or their toxins which are known or reliably believed to cause disease in humans or other living organisms.

	II. Waste which contains or is contaminated with a medicine that contains a biologically active pharmaceutical agent.<br><br>III. Sharp waste, or a body fluid or other biological material (including human and animal tissue) containing or contaminated with a dangerous substance as defined by EC No 1271/2008 (The Classification, Labelling, and Packaging Regulation).<br><br>Not all waste generated in clinical settings qualifies as "clinical waste." For example, packaging from disposable gloves is recyclable rather than hazardous. Clinical waste is classified based on its potential risk to human health and the environment, determining its required treatment and disposal methods. High-risk waste necessitates high-temperature incineration (HTI), while lower-risk waste, including that from infected patients, can undergo alternative treatment (AT) such as sterilisation. Offensive waste (OW), which lacks hazardous properties, can be managed similarly to municipal waste, often through conventional energy-from-waste (EfW) processes.
**Waste disposal and processing: waste segregation guidance**[16]	i. Black bags - General waste: to be used like a household bin. E.g. non-recyclable items such as coffee cups, tissues, greasy food packaging, and small amounts of food and glass.

	ii. Clear bag - Dry mixed recycling: these are for food and drink tins, cans, bottles, paper, cardboard and general mixed plastics. E.g. soft drink bottles, milk cartons, yoghurt pots, margarine tubs and soap bottles.<br><br>iii. Yellow and Black/Tiger stripe bags – Offensive waste: these are for waste that has been used to treat non-infectious patients. E.g. nappies, sanitary products, dressings and non-infectious PPE.<br><br>iv. Orange bags – Clinical infectious waste: these are for waste that's been used to treat suspected or known to be infectious patients. This includes PPE, infectious dressings and plasters and infectious swabs.<br><br>v. Yellow bags – Clinical infectious contaminated waste: these are for infectious waste that has been contaminated with medicine and/or chemicals. This includes infectious medically contaminates diagnostic kits, IV bags, infectious swabs contaminated with medicine and/or chemicals.<br><br>Yellow and Purple Strips bags – Cytotoxic/Cytostatic waste: These are for items that have been contaminated by cytotoxic and cytostatic waste including IV bags, PPE and dressings.

Table 4: COREQ (COnsolidated criteria for REporting Qualitative research) Checklist compliance

**COREQ Domain**	**Compliance**
Domain 1: Research Team and Reflexivity	The researchers' experiences and backgrounds allowed them to be both insiders and outsiders in OTs. AA is a surgical registrar and an academic researcher who has conducted research on surgical sustainability and worked in OTs. This allowed him to capitalise on the benefits of being an outsider (part-time clinician who is not based at the observation sites), questioning taken-for-granted assumptions, and an insider in the natural setting who understands the processes and is able to unravel the nuances of behaviours.23 DRL is a consultant academic surgeon with extensive clinical and non-clinical research experience. SJ, CB, PD, TP, and GJ are experienced non-clinical researchers. Their relative unfamiliarity with OTs allowed them to act as outsiders and mitigate any presuppositions.<br><br>To mitigate the impact of our pre-existing beliefs (as clinicians and non-clinicians with knowledge of sustainability research), reflexivity was maintained throughout to enhance transparency and the trustworthiness of findings, and descriptive rather than evaluative language was prioritised. The main ethnographer noted the descriptive observations and transcribed them as soon as possible after collection. These observations were discussed in the bi-weekly team meetings, in which the other authors could challenge these observations and their interpretation. Having both clinicians and non-clinicians on the research team mitigated the effect of pre-existing beliefs. Finally, the discussions with staff were viewed as opportunities to either corroborate or correct our interpretations.<br><br>Early observations demonstrated that nurses comprised the majority of OT staff and that they were most frequently

	engaged in practices directly related to sustainability (e.g., preparation, equipment handling, and waste disposal). Consequently, nursing behaviours were more visible within our dataset. To mitigate potential bias of research team's composition (physicians and non-clinical researchers),interpretations were discussed within a multidisciplinary research team, and opportunistic discussions with staff, including nurses, were used to corroborate and contextualise the observations. This reflexive process aimed to ensure that the findings were grounded in the perspectives of those directly involved in the behaviours observed.<br><br>To become embedded in the operating theatre environment, the researchers donned theatre attire and positioned themselves in a way that did not disturb the surgical procedure. Additionally, the staff were not informed about the specific details of what was being observed, with only the theatre manager being made aware, in order to limit any influence on their behaviours.
<u>Domain 2: Study Design</u>	Purposive sampling was employed in the selection of specialties and hospitals (which were chosen given the primary ethnographer's established familiarity and rapport from his clinical role). This enabled access to various OTs and facilitated an unobtrusive presence, allowing the ethnographer to blend in with the OT staff. The study started with an unstructured investigative approach (grand tour observations[17]), whereby the main ethnographer (AA) and the senior authors observed one operating list (a session) (hereafter, "list") each. The observational template was developed by the research team through an iterative process (see SDC, Table 2).[18] Further observations were then conducted by AA, a senior researcher (PD), and subsequently by SJ. Photographs of the physical environment were taken for documentation and illustration (never of the patients or staff

	members). Field notes were hand-written and transcribed by the observer at the earliest opportunity to ensure accuracy. Behaviours were assessed as sustainable or unsustainable against relevant local and national guidelines and literature outlined in the SDC.
<u>Domain 3: Analysis and Findings</u>	The data were using NVivo software (QSR International, Melbourne Australia), and an inductive-deductive thematic analysis was conducted. Inductive thematic analysis[19] was first employed, utilising interpretative and descriptive coding. AA and CB double coded 50% of the data. The resulting codes were grouped into themes and sub themes to contextualise and describe the influences. Subsequently, the influences were deductively mapped onto TDF domains. The data and analysis were discussed fortnightly with the research team to ensure agreement of codes amongst the research team.

Table 5: Themes and Influences of (un)sustainable practices in Ots, with examples. The Influences are mapped to the TDF Domains.

Themes	Influences	TDF Domains	Example Observation
Infrequent, inconsistent, and deprioritised sustainable practices	Inadequate education and training	Knowledge	Incorrect waste segregation noted. When asked, the nurse did not know the difference between the bins and is not sure what type of waste goes in which bin (O3; N2).
	Inadequate signage and labelling	Environmental context and resources	A nurse opened surgical ties for the scrub nurse. The packaging (can be recycled) did not have disposal and recycling information, so they discarded it in the orange bin (contaminated waste) (O2; N2).
	Low environmental concerns in healthcare setting compared to outside	Memory, attention and decision processes	A consultant explained that they "*don't care about the environment*" and that they believe that people who care about the environment "*don't do their jobs well*" (O4; S1).
Unevidenced precautionary practices	Disgust	Emotions	A nurse put on non-sterile gloves (NSGs) to hold a clean specimen pot so that the scrub nurse can drop a tissue specimen in (O4; N2).
	Fear of contracting infection		An anaesthesia nurse put on NSGs to help the patient into the operating table before being anesthetised. They explained, "*You never know if the patient has HIV, you need to always be protected*" (O2; AN1).
	Fear of transmitting infection	Beliefs about consequences	Uncontaminated NSGs were discarded in the orange bin (contaminated waste). The nurse explained "*NSGs should always go in orange bin as they don't know if they are clean or contaminated".* Even the domestics department (tasked with routine daily and weekly cleaning) always assume they are contaminated (O5; N3).

Themes	Influences	TDF Domains	Example Observation
Habitual practices established early and prompted due to experiences	Automatic (subconscious) in particular contexts	[lack of] Memory, attention, and decision processes	In between operations, 3 nurses were having informal conversations with each other. Two had disposed of their NSGs after wheeling the previous patient to recovery. They started preparing the theatre for the next operation while conversing. One nurse took out a pair of gloves from their pocket, and the other one put on a new pair to move clean equipment in preparation for the next operation. All 3 discarded their gloves in the orange bin while carrying out the informal conversations (O1; N2;N4;N7).<br><br>A theatre manager explained that packaging should be separated into paper and non-paper and discarded in the green (paper) and transparent (non-paper) bins. Later on, they opened an instrument pack and discarded the packaging in the green bin without separating (O4; TM).
	Learning from seniors and peers at the early stages of training	Social influences	The scrub nurse unnecessarily double gloved for an operation. They said, *"I always double glove. It is something I picked up when I was shadowing"* (O4; N2)
	Previous non-clinical experiences	Emotions	Unnecessary NSG use was observed by a nurse. They explained that they wear NSGs for protection and that they do not feel *"comfortable"* not wearing them in OTs. They told a story about how they became afraid of germs, infection-conscious and risk-averse after catching illness from eating in the canteen when they were a student (O6; N1).
	Previous clinical experiences and training	Behavioural regulation	An SpR was observed to double glove unnecessarily. They explained that training in surgery demands rotating between jobs and the experience from previous surgical jobs standardises their practices. They said: *"I always double glove. It's good practice; I was trained in colorectal surgery"* (O5; S2).
	Individual and professional group variation	Social/professional role and identity<br>Social/professional role and identity	As the patient was wheeled in from the anaesthesia room to the operating room, the two nurses wore NSGs to do various tasks which did not require NSGs. Two surgeons handled patient without wearing gloves and the anaesthetist was wearing the same gloves from induction (O4; N1; N2; S1; S2; A1)

Themes	Influences	TDF Domains	Example Observation
			On average, each nurse unnecessarily put on NSGs 3.8 times compared to 0.8 times for surgeons per operation.<br>In a low-risk breast surgery case, one SpR double gloved for the operation while the other SpR and the consultant wore a single pair of sterile gloves (O2; S2; S3).
Striving for ‘efficiency’	Anticipating and preparing for the next potential task (just in case)	Goals	Ten swabs were opened and only 2 were used for an operation. The scrub nurse explained that the consultant did not state how many they needed so they opened the “*minimum number*” to ensure efficiency. They said, “*We were not sure how many swabs she would need for the first operation, so we opened 10*” (each pack contains 5 swabs) (O3; N2).
Leadership and Communication between staff	Awareness of the items needed for the operation	Knowledge	When the brief did not include every instrument, but nurses resorted to the Kardex (a document detailing equipment used by the consultant in each operation) which contained enough information to open the necessary equipment. There was no opened, unused equipment at the end of that list (O2; N2).
	The surgeon leading the brief	Social influences	When the brief was led by a consultant and they stated which instruments to be opened and which to be on stand-by, there were no opened, unused packs at the end of that operation (O5).
	Organisational policies	Environmental context and resources	A nurse manager explained that the best way to change practices is by changing the Trust’s policy as the nursing staff “*will do what they’re told by the Trust*” (O2; NIC)
The physical environment in OTs	Theatre setup	Environmental context and resources	There were only two bins in the operating room: one orange (contaminated clinical waste) and one transparent (dry mixed recyclable clean material). In the preparation room, there was one green (paper only) and one transparent, but no orange. Most packages are disposed of in the orange bin closer to the patients and to the foot end, which is the incorrect bin (O1).

Abbreviations: NSG: Non-sterile Gloves; SpR: Higher Specialty Registrar; O: Observation; S: Surgeon; N: Nurse; AN: Anaesthesia Nurse; A: Anaesthetist; NIC: Nurse-in-charge

Figure 1: Description of the Physical Environment and Patient Journey

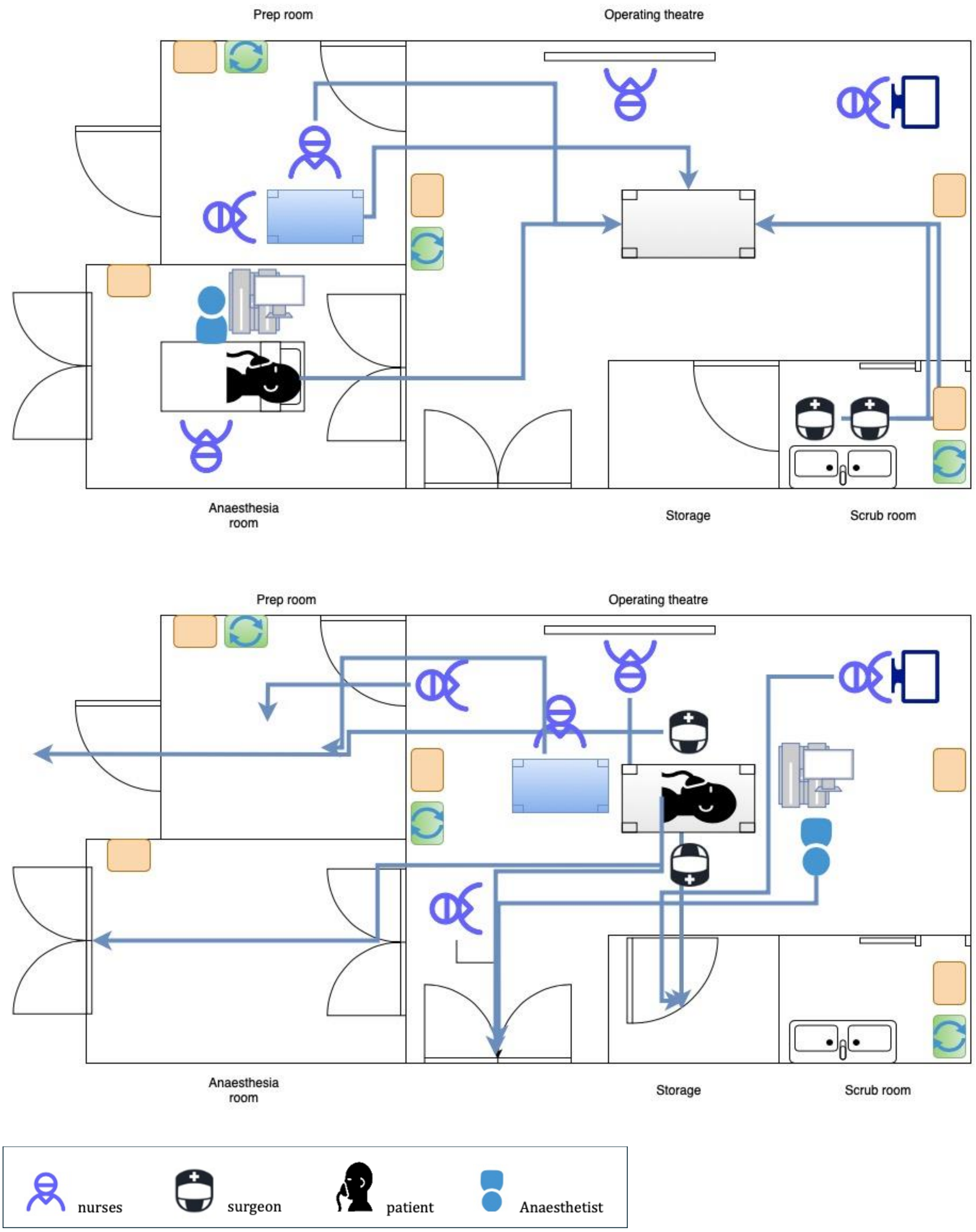

The OT complex includes distinct areas: the anaesthesia room, preparation room, scrub room, and OT. Bin types and locations vary across these spaces, with a notable lack of clinical waste bins and an abundance of contaminated-waste bins. Glove boxes are readily accessible in multiple locations, whereas hand gel dispensers are less so, sometimes almost hidden. In

addition to collective responsibilities (such as patient safety and engaging in the briefing), each member of the surgical team had a distinct role, and specific responsibilities related to the role.

Top: Typical Operating Theatre Complex illustrating layout and staff workflow before moving patient to the operating room for the procedure. Bottom: Operating Theatre layout and staff workflow after moving patient to the operating room and during the procedure. A typical day starts with a team briefing led by a surgeon. The surgical tray is arranged in the preparation room by nursing staff. Anaesthesia and patient positioning occur in the anaesthesia room before the patient is moved to the OT. After the operation, patients are transferred to a recovery room. Between surgeries, OTs are cleaned and sterilised while staff rotate and take breaks. The day concludes with thorough cleaning and sterilisation of theatres in preparation for the next day. In some instances, these processes were adapted, e.g. anaesthetising the patient in the OT.

References

1. LYNCH P, JACKSON MM, CUMMINGS MJ, STAMM WE. Rethinking the role of isolation practices in the prevention of nosocomial infections. *Annals of internal medicine*. 1987;107(2):243-246. doi:10.7326/0003-4819-107-2-243
2. Pratt R, Pellowe C, Wilson J, et al. epic2: National evidence-based guidelines for preventing healthcare-associated infections in NHS hospitals in England. *Journal of Hospital infection*. 2007;65:S1-S59. doi:10.1016/S0195-6701(07)60002-4
3. Wilson J, Breedon P. Universal precautions. *Nursing times*. 1990;86(37):67-70.
4. St Germaine RL, Hanson J, de Gara CJ. Double gloving and practice attitudes among surgeons. *Am J Surg*. Feb 2003;185(2):141-5. doi:10.1016/s0002-9610(02)01217-5
5. Pirie S. Surgical Gowning and Gloving. *Journal of Perioperative Practice*. 2010;20(6):207-209. doi:10.1177/175045891002000603
6. Roebuck A, Harrison EM. Operating theatre etiquette, sterile technique and surgical site preparation. *Surgery (Oxford)*. 2017/04/01/ 2017;35(4):177-184. doi:https://doi.org/10.1016/j.mpsur.2017.01.015
7. Tanner J, Parkinson H. Double gloving to reduce surgical cross-infection. *Cochrane Database Syst Rev*. Jul 19 2006;2006(3):Cd003087. doi:10.1002/14651858.CD003087.pub2
8. Mischke C, Verbeek JH, Saarto A, Lavoie MC, Pahwa M, Ijaz S. Gloves, extra gloves or special types of gloves for preventing percutaneous exposure injuries in healthcare personnel. *Cochrane Database of Systematic Reviews*. 2014;(3)doi:10.1002/14651858.CD009573.pub2
9. Makama JG, Okeme IM, Makama EJ, Ameh EA. Glove Perforation Rate in Surgery: A Randomized, Controlled Study To Evaluate the Efficacy of Double Gloving. *Surgical Infections*. 2016/08/01 2016;17(4):436-442. doi:10.1089/sur.2015.165
10. Laine T, Aarnio P. How often does glove perforation occur in surgery? comparison between single gloves and a double-gloving system. *The American Journal of Surgery*. 2001;181(6):564-566. doi:10.1016/S0002-9610(01)00626-2
11. (CDC) USCfDCaP. Eye Protection for Infection Control. Accessed 25th October 2025, 2025. https://www.cdc.gov/niosh/ppe/eye-safety/infection-control.html
12. England NHSN. National infection prevention and control manual (NIPCM) for England. Updated 29th July 2025. Accessed 25th October 2025, https://www.england.nhs.uk/national-infection-prevention-and-control-manual-nipcm-for-england/version-history/
13. Organization WH. Global guidelines for the prevention of surgical site infection. World Health Organization. Accessed 25th October 2025, 2025. https://iris.who.int/server/api/core/bitstreams/5c422323-5c27-4e6a-812e-dbd76023ba48/content
14. Matsuoka S, Kondo T, Seishima R, et al. Surgical glove perforation during laparoscopic colorectal procedures. *Surgical Endoscopy*. 2022/05/01 2022;36(5):3489-3494. doi:10.1007/s00464-021-08670-0
15. NHS England. NHS Clinical Waste Strategy. NHS Engalnd. Updated 7 March 2023. Accessed 10 February 2025, 2025. https://www.england.nhs.uk/long-read/nhs-clinical-waste-strategy/
16. England N. NHS Property Services | Put waste in its place – know your colours. @nhsproperty. Accessed 10 February 2025, https://www.property.nhs.uk

17. Tjørnhøj-Thomsen T, Hansen HP. Ethnographic Fieldwork. In: Facey KM, Ploug Hansen H, Single ANV, eds. *Patient Involvement in Health Technology Assessment*. Springer Singapore; 2017:149-163.
18. Kitto SC, Chesters J, Grbich C. Quality in qualitative research. *Medical Journal of Australia*. 2008;188(4):243-246. doi:https://doi.org/10.5694/j.1326-5377.2008.tb01595.x
19. Braun V, Clarke V. Using thematic analysis in psychology. *Qualitative Research in Psychology*. 2006/01/01 2006;3(2):77-101. doi:10.1191/1478088706qp063oa
